# Epigallocatechin-3-gallate: a useful, effective and safe clinical approach for targeted prevention and individualised treatment of neurological diseases?

**DOI:** 10.1186/1878-5085-4-5

**Published:** 2013-02-18

**Authors:** Anja Mähler, Silvia Mandel, Mario Lorenz, Urs Ruegg, Erich E Wanker, Michael Boschmann, Friedemann Paul

**Affiliations:** 1 Experimental and Clinical Research Center, a joint cooperation between the Charité University Medicine Berlin and Max Delbrueck Center for Molecular Medicine, Berlin, D-13125, Germany; 2 NeuroCure Clinical Research Center, Charité University Medicine, Berlin, D-10117, Germany; 3 Department of Molecular Pharmacology, Faculty of Medicine, Eve Topf Center of Excellence for Neurodegenerative Diseases Research and Department of Molecular Pharmacology, Faculty of Medicine, Technion, Haifa, 31905, Israel; 4 Medizinische Klinik für Kardiologie und Angiologie, Charité University Medicine Berlin, Campus Mitte, Berlin, D-10117, Germany; 5 Geneva-Lausanne School of Pharmaceutical Sciences, University of Geneva, Geneva, CH-1211, Switzerland; 6 Department of Proteomics and Molecular Mechanisms of Neurodegenerative Diseases, Max Delbrueck Center for Molecular Medicine, Berlin, D-13125, Germany

**Keywords:** Neurological diseases, Predictive and personalised medicine, Targeted prevention, Green tea, Epigallocatechin-3-gallate, Tailored therapy

## Abstract

Neurodegenerative disorders show an increasing prevalence in a number of highly developed countries. Often, these diseases require life-long treatment mostly with drugs which are costly and mostly accompanied by more or less serious side-effects. Their heterogeneous manifestation, severity and outcome pose the need for individualised treatment options. There is an intensive search for new strategies not only for treating but also for preventing these diseases. Green tea and green tea extracts seem to be such a promising and safe alternative. However, data regarding the beneficial effects and possible underlying mechanism, specifically in clinical trials, are rare and rather controversial or non-conclusive. This review outlines the existing evidence from preclinical studies (cell and tissue cultures and animal models) and clinical trials regarding preventive and therapeutic effects of epigallcatechin-3-gallate in neurodegenerative diseases and considers antioxidative vs. pro-oxidative properties of the tea catechin important for dosage recommendations.

## Review

### Introduction

Neurodegenerative disorders show an increasing prevalence in a number of highly developed countries partly attributable to the still increasing life expectancy in these countries but also other still not clearly identified and characterised causal factors. Depending on the disease, sensory, motor, cognitive and autonomic functions might be affected differently. Once diagnosed, neurodegenerative diseases often require life-long treatment, mostly with drugs which are costly and mostly accompanied by more or less serious side-effects. Governments and healthcare providers have to face the emerging health burden of neurodegenerative diseases. Therefore, there is an intensive search for new strategies not only for treating but also for preventing these diseases. At the time of diagnosis, when neurodegeneration has already become evident in terms of symptoms, neurodegenerative cascades are already initiated and neuro-axonal degeneration cannot be reversed with neuroprotective treatments [1]. Green tea and green tea extracts (GTEs) seem to be a promising and safe alternative for targeted prevention and individualised treatment of neurological diseases. However, data regarding beneficial effects and possible underlying mechanism, specifically in clinical trials, are rare and rather controversial or non-conclusive.

The pathogenesis of these diseases includes a number of different processes such as oxidative stress, inflammation, or simply neuronal dysfunction due to neuronal degeneration.

Neuronal tissue is more prone to oxidative damage than other tissues because of (1) its high content of unsaturated fatty acids that are sensitive targets for free radical attack leading to peroxidation, (2) the brain’s high oxygen consumption (20% of total) despite its relatively small (2% of human body) weight, (3) its low antioxidant defence mechanisms, (4) higher iron levels in certain brain regions, and (5) high ascorbate level [2]. Although ascorbate serves an important role as scavenger of free radicals in the human body [3], it can generate free radicals in the presence of Fe^3+^ and Cu^2+^[4].

Tea is the most widely consumed beverage after water. Green tea preparation precludes the oxidation of leaf polyphenols which are thought to contribute to the health-promoting effects. Tea polyphenols, known as catechins, usually account for 30% to 42% of the dry weight of the solids in brewed green tea. The four major catechins (flavan-3-ols) are (−)–epigallocatechin-3-gallate (EGCG), (−)-epigallocatechin (EGC), (−)-epicatechin-3-gallate (ECG), and (−)epicatechin (EC). EGCG represents the most abundant one of tea catechins (50% to 80% of total catechins) [5-7]. These four catechins act as potent antioxidants via direct scavenging of reactive oxygen and nitrogen species (ROS and RNS), induction of defence enzymes and binding and chelating of divalent metals, such as copper and iron [8].

**Figure 1:**
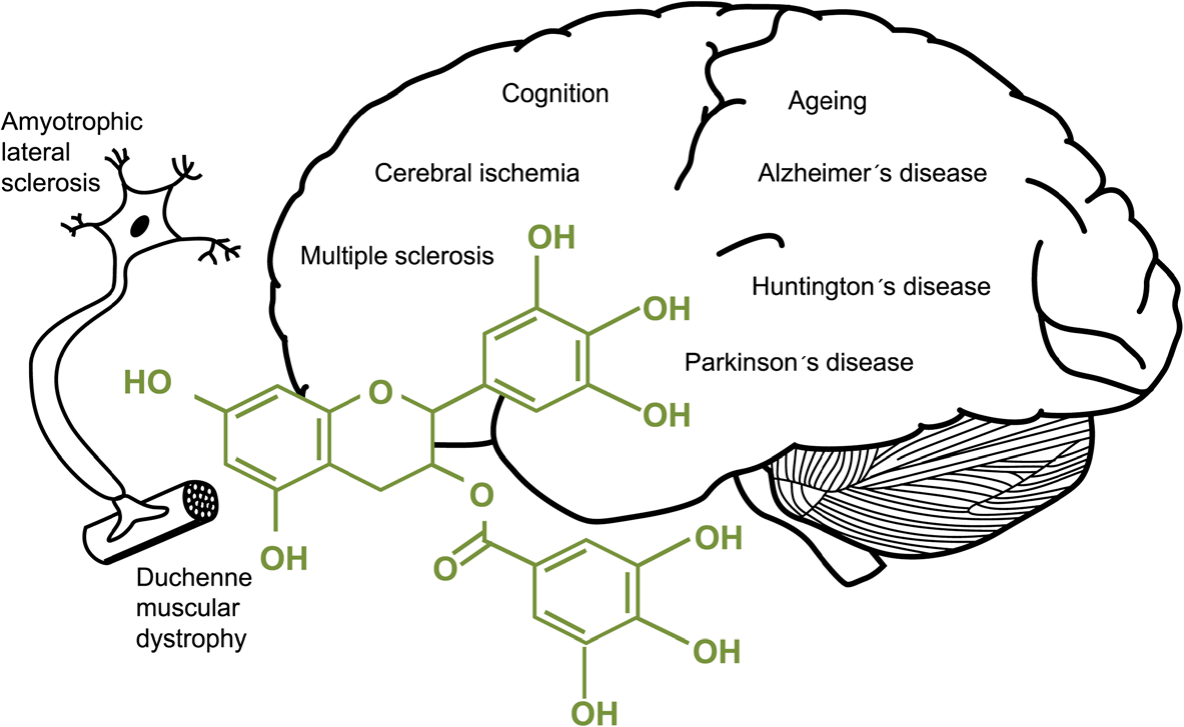
Neurological conditions positively affected by epigallocatechin-3-gallate.

**Figure 2:**
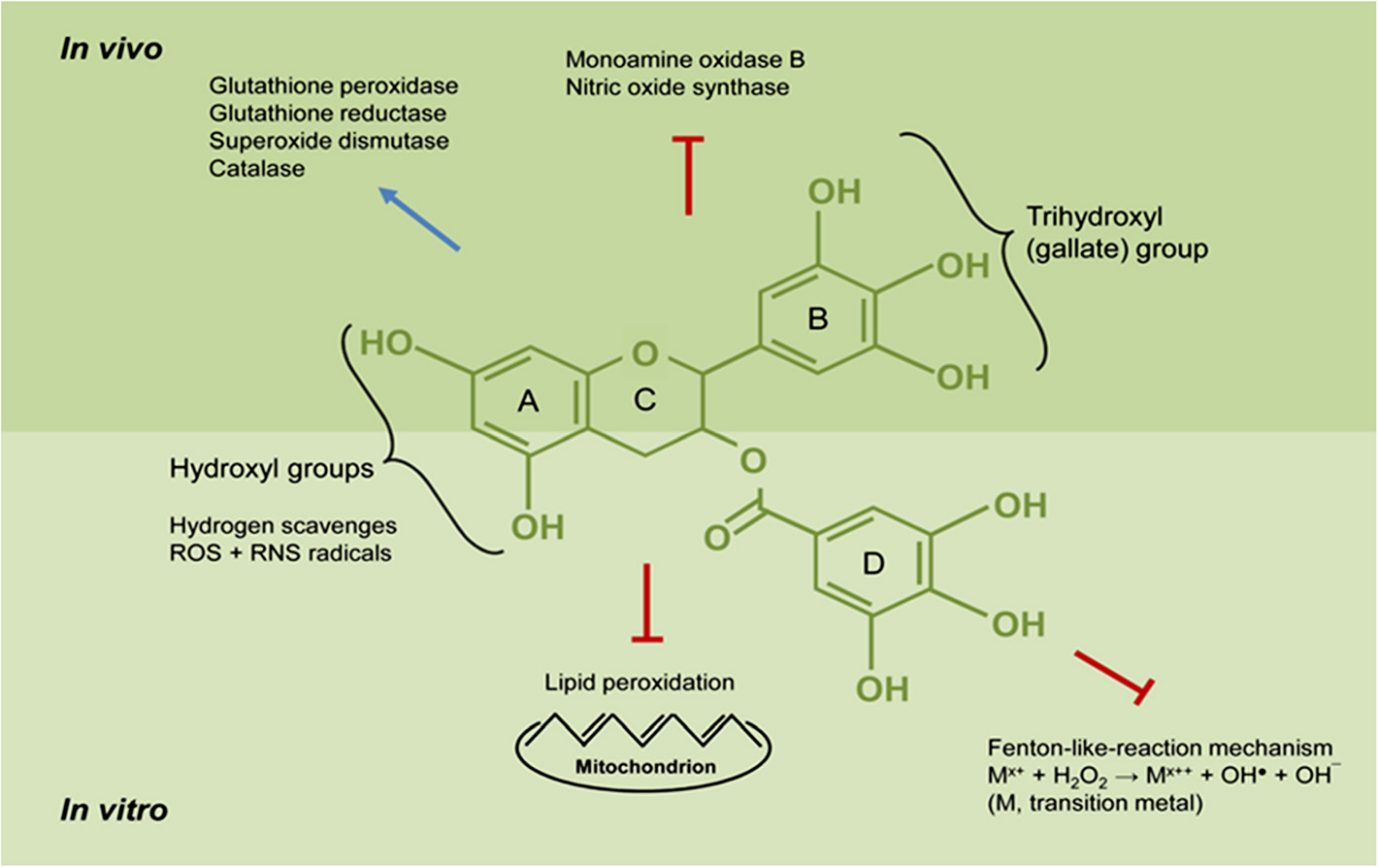
Proposed neuroprotective mechanisms of epigallocatechin-3-gallate.

This review provides information for both basic scientists and clinician scientists in order to encourage translational approaches that address the issues discussed. Undertaking efforts to facilitate translational research with respect to predictive, preventive and personalised medicine in neurodegenerative diseases was recommended recently [1]. The review should meet both: (1) outlining the existing evidence from preclinical studies (cell and tissue cultures and animal models) and clinical trials regarding preventive and therapeutic effects of EGCG in neurodegenerative diseases (Table 1, Figure 1) and (2) considering antioxidative vs. pro-oxidative properties of EGCG important for dosage recommendations. The most important neurodegenerative disorders will be covered.

**Table 1:** Effects of epigallocatechin-3-gallate

**Neurological disease**	**Preclinical studies**	**Epidemiological and clinical studies**
Multiple sclerosis	Inflammation, proliferation and TNF-α secretion of T cells ↓ in EAE mice [9]	Effects on T2 lesions in brain MRI in RRMS? (NCT00525668)
	Combination with glatiramer acetate: cell death ↓ and neuronal outgrowth ↑ in primary neurons, disease severity ↓ in EAE mice [10]	Effects on brain atrophy in progressive forms? (NCT00799890)
	Th1 and Th17 ↓, Treg ↑ in EAE mice [11]	Metabolic effects in MS patients? (NCT01417312)
Alzheimer’s disease	Aβ-induced death of hippocampal cells ↓ [12]	Effects on the course of AD in 50 early stage patients? (NCT00951834)
	α-secretase activity in Alzheimer transgenic mice ↑ [13]	
	Recovery of Aβ-induced memory dysfunction in mice [14]	
	Protection of microglia cells from Aβ-induced ↑ iNOS expression and NO production [15]	
	Direct conversion of fibrillar species into benign protein aggregates [16]	
Parkinson’s disease	Lipopolysaccharide-induced microglial activation ↓ [17]	Inverse relation of tea drinking and PD in Chinese [18], Western Washington [19], Finish [20], and Israelian [21] cohorts
	Protection of PC12 cells against 6-hydroxydopamine-induced apoptosis [22]	
	Attenuation of MPP+-induced ROS production in PC12 cells [23]	Safe and efficient in *de novo* PD patients? (NCT00461942)
	Protection against striatal dopamine depletion and neuronal loss in MPTP mice [24]	
	Loss of dopaminergic neurons ↓, nNOS expression ↓ in MPTP mice [25]	
	No behavioural improvements in 6-hydroxydopamine-lesioned rats [26]	
	Attenuation of increased iNOS expression in MPTP mice [27]	
	Inhibition of levodopa methylation by catechol-*O*-methyltransferase [28]	
Huntington’s disease	Inhibition of huntingtin protein aggregation in yeast and fly models of HD [29]	Efficient (changes in cognitive decline) and tolerable (1,200 mg/day for 12 months) in HD patients? (NCT01357681)
	Memory impairment ↓ in 3-NP-treated rats, glutathione level of neuronal cells ↑ [30]	
Duchenne muscular dystrophy	Elongator digitorum longus muscle necrosis ↓ in *mdx* mice [31]	Safe and tolerable in DMD boys? (NCT01183767)
	Hind limb muscle necrosis ↓, muscle force and fatigue resistance ↑ in *mdx* mice [32]	
	Creatine kinase and oxidative stress ↓, improved histology, utrophin ↑ in *mdx* mice [33]	
	Glutathione synthesis ↑ in muscle cells cultured from *mdx* mice [34]	
	Muscle aerobic metabolism ↑ in combination with endurance training in *mdx* mice [35]	
	Muscle pathology in regenerating fibres ↓ in *mdx* mice [36]	
Amyotrophic lateral sclerosis	Delayed symptom onset, prolonged life span, attenuated death signals in ALS mice [37]	
	Protection of motor neurons, microglial activation ↓ in ALS mice [38]	
	Protection of motor neurons against THA-induced toxicity in rat spinal cord explants [39]	
Cerebral ischaemia	Ischaemia-reperfusion brain injury↓ in gerbils [40-42], rats [43-51] and mice [52,53]	≥3 Cups of tea/day decrease the risk of stroke [54]

**↓** decreased, **↑** increased.

### EGCG: bioavailability and possible therapeutic targets

There is only limited data on the bioavailability of EGCG in the brain, an important prerequisite for its neuroprotective effects. A single, very high oral EGCG dose (500 mg/kg body weight) to rats yielded EGCG concentrations of 12.3 nmol/mL in plasma and 0.5 nmol/g in brain (measured by CL-HPLC) [55]. A single administration of ^3^H] EGCG into the stomach of mice leads to significant amounts of radioactivity in the brain - thought to consist of EGCG itself and its metabolites in free and protein-bound forms. A second administration 6 h later increased brain’s radioactivity above the level measured after the first dose. This finding suggests that EGCG obviously accumulates in the brain when given repeatedly [56]. However, after drinking 250 ml green tea by six human subjects, HPLC-MS analysis revealed that flavan-3-ol methyl-glucuronide and sulfate metabolites appeared in the bloodstream but did not pass through the blood-cerebrospinal fluid barrier [57]. The discrepancy between animal and human data might originate from (1) the very high dose of EGCG applied in the animal model versus the single, rather small dose contained in one cup of tea, (2) the time point (2 h after drinking) chosen for the lumbar puncture in the clinical study, and (3) an apparently more rapid catechin metabolism in humans. Catechins and epicatechin pass the blood brain barrier as shown by *in vivo* microdialysis of rat hippocampus following intravenous application of these basic monomer units of the flavanols [58].

Once in the brain, EGCG can influence numerous processes. Possible underlying mechanisms derived from *in vitro* and *in vivo* studies have been reviewed by Mandel et al. [59]. Briefly, EGCG acts as a powerful hydrogen-donating radical scavenger of ROS and RNS and chelates divalent transition metal ions (Cu^2+^, Zn^2+^ and Fe^2+^), thereby preventing the Fe^2+^-induced formation of free radicals *in vitro*. Among 12 polyphenolic compounds, EGCG most potently inhibited Fe^2+^-mediated DNA damage and iron ascorbate-promoted lipid peroxidation of brain mitochondrial membranes. *In vivo*, EGCG increased expression and activity of antioxidant enzymes, such as glutathione peroxidase, glutathione reductase, superoxide dismutase (SOD) and catalase but inhibits pro-oxidative ones, such as monoamine oxidase (MAO)-B and nitric oxide synthase (NOS) [59].

ROS and RNS are well recognised for being both deleterious and beneficial. Overproduction of ROS results in oxidative stress that damages membrane lipids, proteins and DNA. In contrast, beneficial effects occurring at low/moderate concentrations of ROS/RNS involve anti-infective processes, various cellular signalling pathways and induction of a mitogenic response [60]. ROS are by-products of cellular, mainly mitochondrial, oxidative metabolism. They are formed by enzymes that reduce oxygen, such as MAO, xanthine oxidase, nicotinamide adenine dinucleotide phosphate (NADPH) oxidases and NOS. The ‘primary’ oxygen-derived ROS is the superoxide anion radical (O_2_^·−^) capable of further reactions with other molecules to generate ‘secondary’ ROS, such as hydrogen peroxide (H_2_O_2_), hydroxyl radical, and singlet oxygen [61,62] or RNS by reacting with nitric oxide (NO·) to peroxynitrite anion (ONOO^−^). Within the brain, NO is produced by cell type-specific NO synxthases, i.e. neuronal (nNOS), endothelial (eNOS) and inducible (iNOS) - the latter being specific for glial cells. NO is essential for controlling cerebral blood flow and neurotransmission and is involved in synaptic plasticity, modulation of neuroendocrine functions, memory formation and behavioural activity. However, NO does not only exert protective effects in the central nervous system (CNS) but also mediates tissue damage [63] because of its reaction with O_2_^·−^-yielding OONO^−^[64], an ion that can nitrosylate tyrosine or cysteine residues in proteins [62]. The hydroxyl and trihydroxyl (gallate) groups of EGCG appear important for scavenging physiologically relevant ROS/RNS (Figure 2) [65].

Ferric ion (Fe^2+^) is an essential cofactor for many proteins involved in neuronal function. However, increasing evidence suggests that Fe^2+^ accumulation in the brain is pathologically relevant in CNS disorders. During ageing, total Fe^2+^ concentration increases in some brain regions that are involved in the pathogenesis of degenerative diseases, such as Alzheimer’s, Parkinson’s and Huntington’s disease. This Fe^2+^ accumulation obviously fosters the production of ROS [66], namely the highly reactive OH·, which attacks a large number of functional groups of the biomolecules in neurons. By chelating redox-active transition metal ions, the gallate groups of EGCG are thought to inhibit the Fenton-like-reaction mechanism [62]. Thus, the formation of highly reactive hydroxyl radicals (OH·) is inhibited. Consequently, polyunsaturated fatty acids in, for example, mitochondrial membranes are protected from lipid peroxidation (Figure 2).

To maintain normal cell physiology, enzymatic antioxidant mechanisms counteract ROS formation. Key players within these mechanisms are SOD, catalase and glutathione peroxidase, as well as enzymes involved in the recycling of oxidised glutathione, such as glutathione reductase and glucose-6-phosphate dehydrogenase which is a key enzyme within the pentose phosphate pathway for generating NADPH [67]. In aged rat and adult mouse brain, EGCG augmented the activities of these antioxidative enzymes (Figure 2) [24,68]. EGCG also inhibited the activity of pro-oxidative enzymes such as MAO-B and NOS. MAO–B catalyses the oxidative deamination of many amine neurotransmitters in the brain thereby producing H_2_O_2_. Lin et al. first reported that EGCG supplementation decreases brain MAO-B activity in adult rats [69]. Furthermore, EGCG inhibited iNOS in lipopolysaccharide-treated microglia cells [17] and in a mouse model of Parkinson’s disease [27] and suppressed nNOS in the striatum of mice [25].

There are two major subgroups of glial cells in the brain: the macroglia (astrocytes, oligodendrocytes and ependymal cells) and the microglia which are resident macrophages of the brain [70]. Chronic neurodegenerative disorders are characterised by activation of microglia in the affected neural pathways. Activated microglia is a source of oxidants, prostanoids and inflammatory cytokines that promote neuronal damage which in turn sustains microglial activation [71]. Thus, agents that down-regulate microglial activation could have favourable effects on the course of diseases accompanied by neurodegeneration and inflammation. There is evidence that EGCG counteracts microglial activation in Parkinson’s [17,72,73] and Alzheimer’s diseases [13] and amyotrophic lateral sclerosis [38].

### Therapeutic efficacy of EGCG in neurological disorders and ageing

#### Multiple sclerosis

An important mechanism that underlies the major health benefits of consuming EGCG, such as its anticancer and anti-inflammatory properties, is its suppressive effect on the growth of different cell types. T cells are highly active cells and an effective T cell-mediated immune response depends on rapid T cell expansion. Physiological levels (0.5 to 10 μM) of EGCG inhibited the proliferation of primary T cells from mice. This effect was mediated by inhibition of cell cycle progression and cell division [74]. Thus, EGCG may exert an effect on autoimmune and inflammatory diseases that involve excessive T cell activation, such as multiple sclerosis (MS). MS is an autoimmune inflammatory disorder of the CNS in which focal lymphocytic infiltration not only leads to demyelination but also axonal and neuronal damage in both the grey and the white matter [75,76], resulting in accrual of neurological disability during the course of the disease and measurable atrophy of the brain, the spinal cord and the retina [77-87]. Women who are more frequently affected than men mainly experience disease onset during their childbearing age [88,89]. The cause of the disease is unknown; however, both genetic susceptibility and environmental factors, such as vitamin D deficiency, ultraviolet B radiation exposure, smoking and Epstein-Barr virus infection, have been shown to play a role in disease aetiopathogenesis [90-103]. Neurological disability can occur in any functional system of the CNS and manifests with impaired vision, muscle weakness, ataxia, spasticity, gait abnormalities, sensory disturbances and bowel and bladder dysfunction. A substantial number of patients also suffer from fatigue, cognitive impairment and sleep disorders, all of which may have a considerable negative impact on the quality of life and vocational status [104-108]. There is no cure for MS; injectable disease-modifying drugs such as beta-interferons and glatiramer acetate have a moderate impact on relapse rate and measures of disease activity on brain magnetic resonance imaging (MRI) with a favourable safety profile [109,110]. Newer drugs such as natalizumab and fingolimod are more effective, however, at a price of more severe side effects such as progressive multifocal leukoencephalopathy, macular oedema [111-116] or cardiac side effects [117-119]. Also mitoxantrone, an immunosuppressive compound given as escalation therapy in relapsing-remitting or progressive MS, exhibits potentially life-threatening side effects such as leukaemia or cardiotoxicity that limit its application in MS patients [120-125]. Moreover, none of the aforementioned drugs has proven neuroprotective properties. In an animal model of human MS with experimental autoimmune encephalomyelitis (EAE) [126-129], oral application of EGCG prevented and reversed the disability and inhibited myelin-specific inflammatory responses. This effective protection against relapsing CNS autoimmune disease resulted in a favourable long-term clinical outcome. Furthermore, EGCG reduced axonal damage and neuronal cell death by directly targeting ROS formation. Decreased proliferation of human CD4^+^ T cells incubated with EGCG could be linked to its interference with cell cycle, NF-κB activation and protein degradation pathway (proteasome) [9]. Combined application of EGCG and glatiramer acetate [130-133] synergistically reduced cell death and promoted axonal outgrowth of primary neurons. These effects could be translated into the EAE model in which diminished clinical disease severity was associated with reduced CNS inflammation. Since no unexpected adverse events occurred when the two substances were applied together [10], their use seems promising in clinical trials with MS patients. These findings were recently confirmed and expanded by showing that EGCG dose-dependently ameliorated clinical symptoms and delayed disease onset in mice with EAE, which was well associated with reduced inflammatory infiltration and demyelination in the CNS. The mechanism underlying these effects was altered regulation of CD4^+^ T cell subsets (Th1/Th17 downregulation, Treg up-regulation) [11].

Meanwhile, clinical trials investigate the effects of EGCG on (1) the number of T2 lesions in brain MRI in patients with relapsing-remitting MS (ClinicalTrials.gov identifier: NCT00525668) and (2) brain atrophy in progressive forms of MS (ClinicalTrials.gov identifier: NCT00799890).

Muscle weakness and excessive muscle fatigue [134-136], commonly experienced by MS patients, lead to a reduced physical activity level in the course of the disease. Sufficient energy production and/or supply are mandatory for efficient muscle performance. Owing to the fact that EGCG increases postprandial lipid oxidation in overweight/obese male volunteers [137] and the importance of lipid oxidation to fuel muscle’s energy metabolism, metabolic effects of EGCG in MS patients are currently being investigated (ClinicalTrials.gov identifier: NCT01417312).

#### Neuromyelitis optica

For a long time, neuromyelitis optica (NMO, Devic’s syndrome) has been considered as a rare variant of MS. However, with the detection of a highly specific serum biomarker, an autoantibody targeting the most abundant astrocytic water channel aquaporin-4 (AQP4), in patients with NMO but not MS or other inflammatory or neurological diseases, it has become clear that NMO is a disease entity distinct from MS despite a considerable overlap in clinical presentation and paraclinical findings [138-148]. Although commercial availability of tests for AQP4 antibodies has facilitated differential diagnosis, a considerable number of NMO patients are still misdiagnosed with MS despite emerging evidence for differences in cerebrospinal fluid, MRI and OCT findings [149-156]. A timely and correct diagnosis, however, is of utmost relevance, as some of the classic immunomodulatory drugs given in MS such as beta-interferons, natalizumab and presumably also fingolimod, are ineffective or even harmful in NMO [157-165]. NMO is usually treated with azathioprine, rituximab, mycophenolate mofetil or mitoxantrone [166-171]. Very recently, the role of T cells in the autoimmune disease process in NMO has come into focus of research [172-174]. In addition, neutrophils are also apparently a major player in the pathogenesis of tissue damage [175,176]. Thus, from a pathophysiological standpoint, EGCG with its inhibitory effect on proliferation of T cells and neutrophils [177,178] could theoretically serve as therapeutic agent in NMO, preferably in combination with established immunosuppressive drugs. However, clinical trials are lacking.

#### Alzheimer’s disease

Alzheimer’s disease (AD) is a neurodegenerative disease characterised by progressive impairment of cognitive function and loss of memory in association with widespread neuronal death [179]. Neuronal loss in AD is accompanied by the deposition of amyloid beta protein (Aβ) in senile plaques. ROS-induced lipid peroxidation has been suggested to play an important role in Aβ-mediated neurotoxicity. In cultured hippocampal neuronal cells, EGCG prevented Aβ-induced cell death and lipid peroxidation through its antioxidant property. The fundamental process of Aβ peptide formation is the proteolysis of amyloid precursor protein (APP). APP proteolytic products arise from the coordinated action of α-, β-, and γ-secretases. In the amyloidogenic pathway, Aβ peptides are produced by the initial action of α-secretase. Conversely, the non-amyloidogenic pathway involves events indicative of α-secretase activity. Because intracellular APP is limited, these pathways are believed to compete for their substrate. In cell culture, EGCG promotes the generation of the non-amyloidogenic soluble form of APP via a protein kinase C-dependent activation of α-secretase [12,179,180] and in Alzheimer transgenic mice; it reduced cerebral amyloidosis through promotion of α–secretase activity [13]. Furthermore, it was found that EGCG markedly reduced secreted Aβ levels in the conditioned medium of Chinese hamster ovarian cells, overexpressing the ‘Swedish’ mutated APP (CHO/ΔNL) [181] and in primary neuronal cells derived from transgenic mice bearing the APP Swedish mutation [13]. Consequently, compounds that alter proteolytic cleavage of APP (by enhancing α-, or inhibiting β- and γ-secretase activities) thereby reducing Aβ peptides are interesting therapeutic options for AD treatment [182]. EGCG administration via drinking water recovered Aβ-induced memory dysfunction in mice. The underlying Aβ reduction was facilitated through increased α- and decreased β- and γ–secretase activities (via ERK/NF-κB pathway inhibition) respectively [14].

Oxidative stress has been suggested to play a causative role in the pathogenesis and progression of AD [183]. Aβ accelerates neurodegeneration by activating microglia, the resident macrophages in the CNS, which in turn exert a cytotoxic effect on neurons by releasing ROS and RNS. EGCG protected BV2 microglia cells from Aβ-induced cytotoxicity and apoptotic cell death by suppressing iNOS expression and subsequent NO production, inhibiting intracellular peroxynitrite accumulation and augmenting intracellular glutathione levels [15].

However, EGCG does not only influence the amyloid formation process via modulation of cellular signal transduction pathways and ROS production but also directly converts fibrillar species into benign protein aggregates [16].

To date, these experimental data have not been confirmed in patients with AD. However, investigations of drug and non-drug interventions are needed. They should be specifically designed to meet the needs of people in the early stages of dementia [1]. An ongoing clinical trial is investigating if EGCG positively affects the course of AD as assessed by Alzheimer’s Disease Assessment Scale-Cognitive Subscale (ADAS-Cog) in early state AD patients co-medicated with acetylcholine esterase inhibitors (ClinicalTrials.gov identifier: NCT00951834).

#### Parkinson’s disease

Parkinson’s disease (PD) is an age-related disorder characterised by progressive degeneration of dopaminergic neurons in the substantia nigra leading to resting tremor, rigidity, bradykinesia or slowness, gait disturbance and postural instability [184].

Microglial activation is believed to play a pivotal role in the selective neuronal injury associated with PD [72,73,185-188]. In an *in vitro* study using dopaminergic mesencephalic cells from rat brain, EGCG potently inhibited NO production and tumour necrosis factor-α (TNF-α) release from lipopolysaccharide-activated microglia. Since activated microglia is the cardinal donor of free radicals and inflammatory factors in the brain, this *in vitro* inhibition could be a reasonable explanation for EGCG-mediated neuroprotection *in vivo*[17].

Pheochromocytoma (PC12) cells from rat adrenal medulla, characterised by catecholamine synthesis, metabolism and transport [189], are used as a cellular model of PD. A GTE [190] or EGCG (at an extreme high concentration of 200 μM) [22] exerted protective effects against 6-hydroxydopamine (OHDA)-induced PC12 cell apoptosis and death. A recent study also used PC12 cells to investigate the effects of EGCG on 1-methyl-4-phenyl-pyridine (MPP^+^)-induced cell injury. The authors found that EGCG improved cell viability and attenuated MPP^+^-induced intracellular ROS formation via the SIRT1/PGC-1α signalling pathway [23].

Neurotoxin 1-methyl-4-phenyl-1,2,3,6-tetrahydropyridine (MPTP) produces an irreversible and severe Parkinsonian syndrome that replicates almost all features of PD [191]. Therefore, MPTP is used in animal models to specifically induce dopaminergic neurodegeneration via oxidative stress. In 2001, it was first reported that GTE and EGCG prevented MPTP-induced loss of striatal tyrosine hydroxylase (TH) positive neurons, increased dopamine (DA) turnover and TH activity in mice. As a result of decreased oxidative stress, increased activities of antioxidative enzymes (SOD, catalase) were attenuated by EGCG. In mouse brain homogenates, the pro-oxidative activity of MAO-B was inhibited [24]. NO is thought to play an important role in mediating MPTP-induced oxidative stress. Therefore, another study investigated whether NOS underlies the protection of tea and EGCG against MPTP-induced dopaminergic neurodegeneration in mice. The authors attributed the prevention of dopaminergic neuron loss, DA and DA metabolite depletion and the preservation of striatal TH activity to the inhibition of nNOS expression [25]. Contrary to the MPTP mouse model of PD, in which 2 mg/kg EGCG afforded neuroprotection [24], oral pretreatment with either 1 or 2 mg/kg EGCG did not attenuate dopaminergic neuron loss in the 6-OHDA rat model of PD. In addition, there were no consistent improvements of behavioural measures due to EGCG treatment. This lack of neuroprotection was assumed to be due to different dose and/or length of EGCG administration in the studies. Furthermore, the mechanisms by which MPTP and 6-OHDA exert their neurotoxic effects are different. Unlike MPTP, 6-OHDA did not require glial cells or the dopamine transporter for its activation but spontaneously auto-oxidised to produce damaging ROS [26]. Apart from amounts used and duration of administration, these conflicting results could be due to the different species used. The pharmacokinetic parameters of EGCG in the mouse are more similar to humans in terms of EGCG plasma availability and biotransformation than those of the rat [192], suggesting that findings from mice are more relevant for human studies. Also, the poor bioavailability of oral EGCG in rats [193] could be a reason why similar doses led to different results in the animal models of PD. Higher but non-toxic doses (10 and 50 mg/kg) of EGCG reduced neuronal death and iNOS expression in MPTP model mice giving further evidence for its neuroprotection via NO reduction [27]. Another proposed mechanism, by which green tea polyphenols could protect dopaminergic neurons against MPTP-induced injury, is by their inhibitory effect on the DA-transporter which blocks the uptake of MPTPs active metabolite (MPP^+^) [194].

Levodopa, a precursor in the biosynthesis of dopamine, is considered to be the most effective drug to alleviate motor symptoms in PD patients. Computational molecular modelling showed that EGCG is a potent, non-competitive inhibitor but a poor substrate of catechol-*O*-methyltransferase (COMT) [195]. *In vitro*, EGCG inhibited human liver COMT and, therefore, the metabolic conversion of levodopa to 3-*O*-methyldopa. Rats receiving oral EGCG displayed lower 3-*O*-methyldopa level in plasma and striatum [28].

Owing to the fact that PD is one of the most common neurological diseases, there are a number of epidemiological studies on the influence of environmental factors, such as tea drinking. In 1998, a case-control study with Chinese PD patients and controls showed that regular tea drinking protects against PD [18]. This was confirmed in PD cases and controls from Washington State in whom reduced risk for PD was related with tea consumption (two cups/day) [19]. A large prospective study in the Finish population, which usually drinks little tea, showed a reduced risk of incident PD in subjects who habitually drank three or more cups of tea per day [20]. However, no distinction between green and black tea was made in the questionnaire making it difficult to associate this finding with EGCG. Of note, Western populations are more likely to consume black tea which is fermented and therefore contains less catechins than green tea. The potential relevance was investigated in the Singapore Chinese Health Study. In this prospective cohort study, black tea drinking was inversely associated with PD risk while green tea drinking was unrelated [196]. A retrospective study associated drinking of more than three cups of tea per day with a delayed onset of motor symptoms in Israeli PD patients [21]. In this study, no distinction was made between black and green tea.

Whether these encouraging results from cellular and animal models apply to humans has not been sufficiently investigated in clinical trials yet. However, the application of early interventions at premotor phases of PD is desirable for patients and health care providers. Authors of a Chinese study sought to determine whether green tea polyphenols are effective and safe in the treatment of *de novo* PD patients (who took no antiparkinsonism drugs). Outcome measures were progression of motor dysfunction and cognition, mood and quality of daily life (ClinicalTrials.gov identifier: NCT00461942).

#### Huntington’s disease

Huntington’s disease (HD) is a dominantly inherited neurodegenerative disorder that is caused by an unstable expansion of a CAG repeat within the coding region of the gene that encodes for the protein huntingtin (htt). The mutation results in an elongated stretch of glutamine near the NH_2_ terminus of the protein. HD symptoms mainly occur as adult-onset HD between age 35 and 50 and comprise personality changes, generalised motor dysfunctions, cognitive decline and neuroendocrine disturbances [197,198].

Growing evidence suggests that the misfolding and aggregation of htt is central to HD pathogenesis. The screening of a library of natural compounds identified EGCG and related polyphenols as potent inhibitors of mutant htt exon 1 protein aggregation *in vitro*. Additionally, EGCG modulated misfolding as well as the assembly of oligomers in cell-free assays and reduced both toxicity and aggregate formation in yeast and fly models of HD [29].

3-Nitropropionic acid (3-NP), a fungal-derived neurotoxin that irreversibly inhibits succinate dehydrogenase, is a well-known model to study the pathogenesis of HD. Memory disturbances in rats caused by 3-NP treatment were attenuated by chronic EGCG treatment. Striatal, cortical and hippocampal glutathione levels of the rats were significantly reduced by 3-NP treatment that was reversed by chronic EGCG treatment. These effects were attributed to NOS inhibition as evidenced by the application of NO modulators (l-arginine and l-NG-nitroarginine methyl ester) [30].

A multicenter trial evaluating the efficiency and tolerability of EGCG in HD patients is currently ongoing. Primary outcome measures are changes of cognitive functions (as measured by UHDRS-Cognition composite score of Stroop test, verbal fluency and Symbol Digit Modalities Test) after 12 months maximal daily intake of 1,200 mg EGCG compared to baseline intake (ClinicalTrials.gov identifier: NCT01357681).

#### Duchenne muscular dystrophy

Another disease, in which the generation of ROS seems to play an important pathogenic role, is Duchenne muscular dystrophy (DMD) [199-201]. DMD is a severe X-linked congenital disorder characterised by progressive muscle wasting caused by the absence of the structural protein dystrophin. The pathogenesis of DMD is frequently studied in the dystrophic *mdx* mouse model. Dietary GTE supplementation preferentially protected the elongator digitorum longus muscle rather than the soleus muscle of *mdx* mice from necrosis [31]. Histological examination of leg muscles and functional recordings of the triceps surae muscle contraction showed that GTE and EGCG protected the hindlimb muscle of dystrophic mice from massive necrosis and greatly improved muscle force and resistance to fatigue [32]. Subcutaneous EGCG injections into the backs of *mdx* mice starting immediately after birth (1) reduced the activity of serum creatine kinase to almost normal levels, (2) decreased oxidative stress indicated by the number of lipofuscine granules in muscle fibres, (3) improved the histology of both the fast-contracting diaphragm muscle and the slow-contracting soleus, (4) increased the mean time for the maximum tetanic force to fall by a half (T1/2max) in *mdx* soleus muscles, and (5) increased the amount of utrophin (partially compensating for the lack of dystrophin) formed in *mdx* diaphragm muscle [33]. A study on cultured muscle cells from *mdx* dystrophic mice showed that green tea polyphenols and EGCG offer both immediate antioxidant effects and long-term prevention by stimulating glutathione synthesis and protect against H_2_O_2_ toxicity [34]. The above mentioned findings were recently extended to study different administration routes and dosages of EGCG to find the most effective for limiting the onset of dystrophic lesions in both the same strain of *mdx* mice and assessment methods. Oral administration of 180 mg/kg EGCG daily in the diet for 5 weeks most effectively reduced muscular dystrophy [202].

Endurance training (i.e. voluntary wheel running) and 0.5% GTE in the diet synergistically improved skeletal and cardiac muscle aerobic metabolism and serum antioxidant capacity and decreased lipid peroxidation and cardiac hypertrophy in young *mdx* mice [35]. Prenatal and early dietary intervention with GTE decreased dystrophic muscle pathology potentially by suppressing NF-κB activity in regenerating fibres of *mdx* mice [36].

In light of this considerable body of evidence, clinical studies in DMD patients should be performed. One registered multicenter, prospective, double blind, placebo-controlled randomised pilot study is investigating safety and tolerance of EGCG in boys with muscular dystrophy of the Duchenne type (ClinicalTrials.gov identifier: NCT01183767).

#### Amyotrophic lateral sclerosis

Amyotrophic lateral sclerosis (ALS) is a fatal neurodegenerative disorder in which degeneration of upper and lower motor neurons leads to progressive muscle weakness and atrophy [203,204]. In recent years, non-motor involvement has been increasingly reported in ALS [205-212]; a clinical, pathological and genetic continuum with frontotemporal dementia has been acknowledged [213,214]. Moreover, it became evident that inflammatory mechanisms, including microglial activation, contribute not only to neuronal cell death but may also promote neuronal survival [210,215-218]. Drugs targeting inflammatory pathways showed beneficial effects on survival in transgenic ALS mouse models [219]. The first *in vivo* study on EGCG’s effects on ALS was performed on ALS model (SOD1-G93A) mice. Oral EGCG treatment (>2.9 μg/g body weight) significantly delayed symptom onset, prolonged life span, preserved survival signals and attenuated death signals [37]. The disease onset delaying and survival prolonging effects of oral EGCG treatment in ALS mice were confirmed by another study. EGCG protected the motor neurons and reduced microglia activation and associated iNOS expression, such as NF-κB and caspase-3 [38]. Another widely used model to study ALS employs threohydroxyaspartate (THA), an inhibitor of glutamate transport, to induce motor neuron death in cultured rat spinal cord explants. In this model, EGCG protected motor neurons against THA-induced toxicity, which was accompanied by regulation of glutamate levels in the synaptic cleft, and decreased tissue levels of lipid peroxides [39].

#### Cerebral ischaemia

Stroke is the second most common cause of death and major cause of disability worldwide [220]. Temporary or permanent reduction of blood flow deprives the brain of oxygen and glucose which causes structural damage during ischaemia and reperfusion [43,221]. Neuronal cell death is delayed for several days after ischaemic insult and is restricted to sensitive areas of the brain, such as the hippocampus and the striatum [52]. Possible neuroprotective effects of EGCG on cerebral ischaemia have been studied extensively in animal models. Administration of GTE/EGCG in ischaemia-reperfusion brain injury decreased the extent of neuronal injury in experimental models of stroke in gerbils [40-42], rats [43-51] and mice [52,53].

Proposed protective actions of GTE/EGCG are (1) increase of nNOS and eNOS but decrease of iNOS expression [43,47,49], (2) inhibition of matrix metalloproteinase-9 activity (which decreases degradation of matrix components of basement membranes thereby maintaining cerebral vasculature integrity) [52,53], (3) amelioration of synaptic transmission [51], and (4) stimulation of 67LR, increased generation of ROS via NADPH oxidase and subsequent activation of PKCε [221]. One of the above mentioned animal studies observed a >50% increase of intracerebral haemorrhages after EGCG treatment. Consequently, EGCG might not be an appropriate intervention for the acute treatment of ischaemic stroke [46].

A meta-analysis revealed that, regardless of their country of origin (China, Japan, Finland, The Netherlands, Australia and USA), individuals consuming ≥3 cups of tea per day had a 21% lower risk of stroke than those consuming <1 cup per day [54]. To the best of our knowledge, there are no clinical studies addressing effects of EGCG in human stroke.

#### Cognitive behaviour

Some evidence from animal and human studies indicates a possible contribution of EGCG in the prevention of cognitive decline. Increased release of glutamate from CNS neurons is considered to be an indicator of maintenance of cognitive functions, such as learning and memory [222,223]. On isolated nerve terminals prepared from rat cerebral cortex, EGCG potently facilitated evoked glutamate release by enhancing Ca^2+^ entry through voltage-dependent Ca^2+^ channels, rather than any upstream effect on nerve terminal excitability. This release was linked to the activation of protein kinase C (PKC) and cytoskeleton disassembly [224].

Long-term (26 weeks) administration of green tea catechins to rats improved their performance in radial maze tasks and hippocampal levels of lipid peroxides. Consequently, green tea catechins may be involved in protecting against neuronal degenerative stress and in the accumulation of lipid peroxides and ROS [225]. EGCG treatment of rats before and after traumatic brain injury eliminated and/or absorbed free radicals induced by brain injury. As a result, neuronal cell degeneration and apoptotic cell death around the damaged area were inhibited, and brain dysfunction was improved [226]. Fatigue is an overwhelming sense of tiredness or lack of energy, affecting both mental and physical domains. In a rat model of load-induced chronic fatigue syndrome, EGCG restored all behavioural and biochemical alterations produced by chronic fatigue [227].

There are, albeit limited, data on the effects of EGCG on human cognition. A cross-sectional analysis of a self-administered questionnaire showed that higher green tea consumption is associated with less prevalent cognitive impairment in elderly Japanese subjects [228]. In a double-blind, placebo-controlled, balanced crossover study, the effects of oral doses of EGCG on cerebral blood flow in the frontal cortex during tasks that activate this brain region and cognitive performance were investigated. A single dose of 135 mg EGCG reduced cerebral blood flow during task performance. However, this was not associated with any significant modulation of either cognitive performance or mood [229].

#### Ageing

Ageing is the progressive accumulation of changes over time that is associated with, or responsible for, increasing susceptibility to disease and death [230]. Oxidative stress has been associated with both the ageing process and the development of age-dependent tissue degenerative pathologies.

The nematode *Caenorhabditis elegans* is a tiny roundworm of 1 to 2 mm in length that colonises various microbe-rich habitats, in particular, decaying plant matter. Due to its rapid lifecycle, it is one of the major model organisms in ageing, genetic, molecular and other biological observations [231-234]. EGCG attenuated age-related pharyngeal contraction decline, moderately alleviated Aβ-induced behavioural pathologies and sustained an increased chemotactic behaviour. However, there were no significant increases in mean or maximal life span due to EGCG feeding [235]. This was confirmed by another study that found no significant longevity-extending effects of EGCG in *C. elegans* under normal culture conditions but with extension of life span by EGCG under heat and oxidative stress [236]. In another investigation on wild-type N2 and transgenic strains of *C. elegans*, EGCG administration increased the mean lifespan, inhibited heat shock protein expression and decreased intracellular H_2_O_2_ levels [237].

A systematic study on the effects of EGCG on ageing and ageing promoting factors, such as ROS accumulation, mitochondrial integrity and antioxidative enzyme activity in human fibroblasts, directly linked mitochondrial integrity to the efficiency of the antioxidant defence system in the ageing process [238]. In mice in which ageing had been induced by d–galactose, EGCG improved learning and memory functions, increased SOD and glutathione peroxidase activities and decreased the hippocampal malondialdehyde content and neuronal apoptosis [239].

Several neuronal systems, such as dopaminergic, cholinergic and serotoninergic ones, undergo alterations during ageing. Rats supplemented with EGCG had increased cortical neurotransmitter levels (dopamine, acetylcholine and serotonin) and acetylcholine esterase activity and performed better in radial maze experiments when compared to aged-matched controls [240].

### Dose regimen: antioxidative versus pro-oxidative

As green tea/EGCG are becoming more and more popular due to their proposed health benefits, persons with different pathological conditions might consume commercially available supplements that contain catechin concentrations exceeding those of tea preparations by far. Owing to their reducing ability, antioxidant compounds can activate transition metal ions (e.g. Fe^3+^ to Fe^2+^ or Cu^2+^ to Cu^+^), making them behave as pro-oxidants. It has been demonstrated that four common dietary antioxidants (cysteine > ascorbate > EGCG > glutathione), in the presence of copper (cupric sulfate and cupric gluconate) and physiologically relevant levels of H_2_O_2_, can also act as pro-oxidants by producing hydroxyl radicals [61]. Yoshioka et al. reported that tea catechins advanced DNA damage and lipid peroxidation in the presence of Cu^2+^. Tea catechins formed a complex with Cu^2+^ resulting in the formation of hydroxyl radical that, in the case of EGCG, was scavenged by the additional gallate group [241]. Interestingly, a mixture of the four tea catechins (Polyphenon 100) and individual tea catechins differently modulated human cytochrome P450 1A expression. The underlying mechanisms of increased effectiveness of green tea and a catechin mixture compared to single catechins were not elucidated in this study [242].

Under *in vitro* conditions, catechins exerted antioxidant and antiapoptotic properties at low concentrations (1 to 50 μM), whereas at higher concentrations (100 to 500 μM) the reverse was observed [243]. It was demonstrated that GTE and EGCG inhibit cell growth and induce cell death at concentrations of 10 to 20 μM. In murine macrophage and human leukemic cell lines, EGCG increased H_2_O_2_-induced oxidative stress and DNA damage. The oxidant activity of EGCG exceeded that of H_2_O_2_. Therefore, excessive EGCG concentrations could induce toxic levels of ROS *in vivo*[244]. EGCG dose-dependently inhibited the growth of H1299 cells in culture and in xenograft tumours and induced ROS formation both *in vitro* and *in vivo*[245].

In murine and human plasma, EGCG concentrations up to 4 μM were reported after single doses [246-248]. However, repeated doses might lead to higher concentrations with possible deleterious effects. In healthy adult mice fed with diets containing none, moderate (0.15% and 0.3%) or high (1%) *w/w* EGCG for 6 weeks, inflammatory responses were studied. While no influence of moderate EGCG levels was observed, the high dose significantly elevated several pro-inflammatory markers. This was accompanied by significant weight loss without visible toxicity as assessed by the histological examination of several key organs [249].

For several reasons, extrapolation of these animal data to the human situation is difficult, if not impossible. It has to be ensured that patients treated with EGCG benefit from its antioxidative effects and, at the same time, are not harmed by pro-oxidative effects. Factors that could lead to conflicting results between *in vitro* and *in vivo* studies are varying degrees of responsiveness to EGCG in patients (responder vs. non-responder), poor bioavailability (cave:blood sample collection) that is influenced by autoxidation (air contact) or metal ions in food and water (calcium, magnesium and iron), extensive biotransformation in the liver and variations in serum albumin levels. To optimise EGCG bioavailability, capsules should be stored cool and dry, taken at least 30 min before breakfast/dinner with soft water and possibly combined with ascorbic acid, sucrose or fish oil [250]. To avoid potential side effects, clinical trials should include close attention to patients, control of liver enzymes and regular determination of outcome measures.

## Conclusions

EGCG is thought to interfere with several pathways in numerous neurological functions in health and disease. Despite a considerable body of evidence from cell and animal models, there is a lack of epidemiological and clinical studies on potential health benefits of EGCG in patients with the neurological conditions discussed. Within the clinical studies, the number of subjects is mostly rather small and patients are not well phenotyped or standardised regarding disease severity and duration. Green tea preparations and GTEs are also not standardised. Therefore, optimum dose of EGCG for preventing or treating a disease is still a matter of debate. Before EGCG can be recommended as a targeted prevention and individualised treatment to patients, the plasma and brain bioavailability, dose-response effects, safety, tolerability, efficacy and possible interactions with other drugs have to be studied in more detail and in a disease-specific manner. For a number of clinical effects the underlying mechanisms are still unclear. Because EGCG is acting on so many different routes (neuro-endocrine, metabolic, defence and others), studies with an integrated approach are strongly needed. This will be, however, a cost-intensive and time consuming endeavour.

## Competing interests

The authors declare that they have no competing interests.

## Authors’ contributions

AM, MB, and FP drafted the manuscript. SM, ML, UR, and EW completed and critically revised the manuscript. All authors read and approved the manuscript.
